# Informal Caregiving in Patients With Atrial Fibrillation and Multimorbidity: A Cross-European Study of Caregiver Burden, Health-Related Quality of Life, and Caregiver Engagement

**DOI:** 10.1097/JCN.0000000000001289

**Published:** 2025-12-19

**Authors:** Caterina Bosio, Dilara Usta, Caterina Trevisan, Deirdre A. Lane, Guendalina Graffigna

**Affiliations:** 1 Caterina Bosio, PhD, Project Manager, EngageMinds HUB—Consumer, Food & Health Engagement Research Center, Catholic University of Sacred Heart, Cremona, Italy.; 2 Dilara Usta, PhD, RN, RN Postdoctoral Fellow, EngageMinds HUB—Consumer, Food & Health Engagement Research Center, Catholic University of Sacred Heart, Cremona, Italy.; 3 Caterina Trevisan, PhD, Associate Professor, Department of Medicine, University of Padova, Italy; Department of Medical Sciences, University of Ferrara, Italy; Aging Research Center, Karolinska Institutet, Stockholm, Sweden.; 4 Deirdre A. Lane, PhD, Professor of Cardiovascular Health, Adjunct Professor, Department of Cardiovascular and Metabolic Medicine, Faculty of Health and Life Sciences, Institute of Life Course and Medical Sciences, University of Liverpool, United Kingdom; Liverpool Centre for Cardiovascular Science, University of Liverpool and Liverpool Heart and Chest Hospital, United Kingdom; Department of Clinical Medicine, Aalborg University, Denmark.; 5 Guendalina Graffigna, PhD, Full Professor, EngageMinds HUB—Consumer, Food & Health Engagement Research Center, Catholic University of Sacred Heart, Cremona, Italy.

**Keywords:** atrial fibrillation, caregiver, caregiver burden, health-related quality of life, multimorbidity

## Abstract

**Background:**

Atrial fibrillation (AF), often accompanied by multimorbidity, places heavy demands on informal caregivers. Although caregiver burden is recognized in other chronic conditions, little is known about burden, health-related quality of life (HRQoL), and engagement among caregivers of patients with AF across Europe.

**Objectives:**

The aim of the study was to examine caregiver burden, HRQoL, and engagement among informal caregivers of patients with AF and multimorbidity, and to explore interrelationships between these outcomes and caregiving characteristics.

**Methods:**

A cross-sectional online survey was conducted between May 2022 and January 2023 with 179 informal caregivers from Italy, Romania, and Spain. Validated instruments assessed burden (Bakas Caregiving Outcomes Scale), HRQoL (EQ-5D-3L), and caregiver engagement (Caregiving Health Engagement Scale). Group differences were tested using nonparametric analyses, and associations were examined through partial Spearman correlations, adjusting for sociodemographic and caregiving variables.

**Results:**

Participants reported moderate burden and preserved HRQoL in physical domains, but frequent pain/discomfort and anxiety/depression. Engagement was generally low, with most informal caregivers in early or intermediate stages of engagement. Burden was higher among women, younger caregivers, and those in Italy and Romania, while HRQoL was poorer in older caregivers, cohabitants, and those caring for patients with a higher number of comorbidities or reduced mobility. Highly engaged caregivers reported lower burden, better overall health, and less anxiety/depression than low-engaged peers.

**Conclusions:**

Informal caregivers of patients with AF and multimorbidity face psychological strain despite preserved physical functioning. Engagement was a protective factor associated with a lower burden and improved well-being. Fostering engagement and collaboration with professionals may ease strain and support sustainability.

**What’s New and Important**

- Informal caregivers of patients with atrial fibrillation and multimorbidity often maintain physical functioning but experience significant psychological strain, particularly anxiety and depression.
- Informal caregivers with high levels of engagement had lower burden, better perceived overall health, and improved outcomes in the anxiety/depression dimension of health-related quality of life.
- Notable differences emerged by age, sex, cohabitation status, country, daily caregiving time, patient comorbidity, and patient mobility, showing that caregiver burden and quality of life vary across demographic and contextual characteristics.

Atrial fibrillation (AF) is the most common persistent cardiac arrhythmia and is associated with increased hospitalizations, medical costs, and heightened risk of severe complications such as stroke, heart failure, and cardiovascular mortality.^1^ Atrial fibrillation rarely occurs in isolation; multimorbidity is the rule rather than the exception, complicating disease management due to overlapping symptoms, polypharmacy, and increased care complexity.^2,3^ These challenges extend beyond patients and healthcare systems, profoundly affecting informal caregivers, who frequently assume critical roles in daily disease management.^4,5^

Informal caregivers, defined as individuals who provide unpaid support and assistance to relatives, friends, or others with chronic illnesses, disabilities, or age-related conditions, deliver up to 80% of long-term care across Europe and represent a vital component of healthcare systems.^6^ For patients with AF and multimorbidity, informal caregivers manage medication regimens, monitor symptoms, coordinate healthcare appointments, provide emotional support, and often deliver basic care in the home.^7^ While these roles are indispensable, the cumulative demands can adversely affect informal caregivers’ physical and mental health, increasing their risk of burnout, social isolation, and even premature mortality compared with noncaregiving peers.^8,9^ Elevated levels of anxiety, depression, and stress are frequently reported, particularly when navigating the uncertainties of multimorbidity,^10,11^ and caregiving responsibilities may lead to isolation as caregivers prioritize patient needs over personal relationships and activities.^7^

The negative consequences of informal caregiving extend to health-related quality of life (HRQoL). Evidence suggests that caregivers of patients with AF experience reduced HRQoL, particularly when caring demands are long-term, intensive, or associated with comorbid conditions such as diabetes or respiratory disease.^12,13^ Similar patterns are observed among informal caregivers of patients with heart failure, further highlighting the intersection between cardiovascular disease, caregiver burden, and diminished HRQoL.^14^ As anticipated, the cumulative objective and subjective burdens of informal caregiving—combined with cultural expectations and unequal access to support across countries—can discourage caregivers from adapting effectively to their roles.^6^ The deterioration of HRQoL, along with persistent emotional and physical strain, often undermines resilience and may hinder caregivers from maintaining a sustainable balance between caregiving demands and their own well-being.^12–14^ Despite these challenges, a critical area of inquiry is how informal caregivers can preserve engagement in their role, reframing responsibilities and sustaining collaboration with healthcare professionals to ensure continuity of care.^15,16^

The Caregiver Health Engagement Model provides a valuable framework for conceptualizing this process.^16^ It describes caregiving as a multidimensional and evolving journey across 4 domains: (i) emotionally processing the change, (ii) establishing a proactive, balanced stance in the care process, (iii) building effective relationships with healthcare professionals and the broader system, and (iv) managing the care needs of one’s loved ones. Progression within the model ranges from denial, marked by emotional shock and withdrawal from caregiving responsibilities, to balance, where caregivers integrate their role into daily life and achieve collaborative, sustainable engagement with clinicians.^16^

While much of the literature on caregiver burden and HRQoL has centered on informal caregivers of patients with cancer, stroke, heart failure, or Alzheimer’s disease,^14,17–22^ caregivers of patients with AF remain underrepresented, particularly in the context of multimorbidity.^8,12^ Furthermore, the psychological and social dimensions of caregiver engagement, as well as potential cross-cultural differences, remain largely unexplored. To address these gaps, in this study, we aimed to examine the caregiving experiences of European informal caregivers of patients with AF and multimorbidity, focusing on caregiver burden, HRQoL, and caregiver engagement, and exploring the interrelationships between these outcomes.

## Methods

### Study Design, Participants, and Procedure

In this cross-sectional study, we used the data from the work package 4 survey of the AFFIRMO project,^23^ which involved patients and informal caregivers of patients with AF and multimorbidity in 3 European countries: Italy, Romania, and Spain. For the present study, we considered only the data of informal caregivers. In particular, eligible participants were informal caregivers aged 18 years or older who provided care for patients diagnosed with AF and at least one coexisting chronic condition. Exclusion criteria included (i) inability to provide informed consent, (ii) moderate or severe cognitive impairments diagnosed by a healthcare provider (eg, dementia), (iii) the presence of health conditions hindering survey completion, (iv) lack of access to an online platform, (v) providing care for remuneration, and (vi) unwillingness to participate.

A convenience sampling method was employed. Participants were recruited through announcements on the Atrial Fibrillation Association website and by healthcare professionals informing informal caregivers of patients attending clinical appointments at partner hospitals in Italy, Romania, and Spain. Healthcare professionals were approached by email through the project consortium’s professional networks and requested to disseminate the survey among patients attending clinical visits at participating hospitals. Between May 31, 2022, and January 31, 2023, informal caregivers from the involved countries completed an online survey to capture their caregiving experiences. Details on the development of the questionnaire have been reported previously.^24^

### Instruments

#### Characteristics of Informal Caregivers and the Assisted People

Sociodemographic and caregiving variables included age, sex, ethnicity, country of origin, educational attainment, relationship with the assisted person, cohabitation with the assisted person, daily caregiving time commitment, and duration of caregiving. Data on the assisted person, including comorbidities, number of medications, mobility level, and daily activities requiring assistance, were collected to provide a comprehensive context for understanding caregiver experiences.

#### Caregiver Burden

Caregiver burden was assessed with the Bakas Caregiving Outcomes Scale,^25^ a 15-item unidimensional scale designed to evaluate changes in social functioning, subjective well-being, and specific health aspects resulting from caring for a family member with chronic conditions. An additional item broadly evaluates the extent to which a caregiver’s life has been affected by caring for the patient, but it is not included in the total score. The items assess caregivers’ response to the extent of life changes on a 7-point scale, which ranges from −3 (“changed for the worse”) to +3 (“changed for the best”). A value from 1 to 7 is given to each score (where −3 = 1 and +3 = 7), with subsequent summation of the individual item scores. Scores greater than +4 indicate that the caregiver’s life has improved due to caregiving, whereas scores of +4 or less reflect negative perceptions of the caregiving experience. The total score is calculated by summing the individual item scores, yielding a possible range of 15 to 105. A previous reliability assessment of the scale demonstrated strong internal consistency (Cronbach’s α = 0.87),^26^ and the current study exhibited excellent internal consistency, with Cronbach’s α of 0.95.

#### Health-Related Quality of Life

Health-related quality of life was assessed using the European Quality of Life Survey-5 Dimension-3 Levels (EQ-5D-3L) instrument.^27^ It measures HRQoL across 5 domains: (i) mobility, (ii) self-care, (iii) usual activities, (iv) pain/discomfort, and (v) anxiety/depression. The instrument assesses each dimension using 3 levels of severity: no problems, some problems, and extreme problems. The Visual Analogue Scale allows individuals to rate their overall health status, ranging from 0 (“The worst health you can imagine”) to 100 (“The best health you can imagine”), with higher scores indicating better HRQoL.

#### Caregiver Engagement

Caregiver engagement was evaluated using the Caregiving Health Engagement Scale, a validated tool to monitor informal caregivers’ psychological attitudes regarding their participation, competence, and motivation in responding to the care demands of their relatives.^16^ The scale was built on the Caregiving Health Engagement Model, conceptualizing the informal caregiving experience as a dynamic and psychosocial process.^16^ The instrument incorporates 7 items scored on a 7-point scale with a single factor and ordinal structure, enabling caregivers to place themselves in intermediate positions and avoid social desirability bias. The original scale demonstrated good internal reliability (Ordinal α = 0.88), and the present study showed good internal consistency (Cronbach’s α = 0.83).

### Statistical Analysis

Statistical analyses were conducted via IBM SPSS 29 (IBM Corp., Armonk, New York). Categorical variables are represented as frequencies and percentages, while continuous variables are presented as medians with interquartile ranges (IQRs). Normality was assessed using the Shapiro–Wilk test, which yielded a *P*-value <.001, indicating that the data significantly deviated from a normal distribution. Therefore, nonparametric statistical methods were applied in subsequent analyses.

A post hoc power analysis using G*Power 3.1.9.7 was conducted to determine if the study had sufficient statistical power to detect differences between groups with high and low caregiver engagement. Power was calculated using the Mann–Whitney *U* test to compare groups with high and low caregiver engagement, assuming a moderate-to-large effect size (*r* = 0.5),^28^ and a significance level of α = 0.05. For the caregiver engagement groups (n = 124 vs. n = 55), the post hoc power analysis indicated an achieved statistical power of 85.1%. This value exceeds the conventional 80% threshold, confirming that the current study had sufficient power to detect actual differences between engagement groups.

The Kruskal–Wallis test and Mann–Whitney *U* test were used to explore differences in questionnaire scores across various subgroups, including age, sex, country, cohabitation with the assisted person, daily caregiving time, duration of caregiving, number of comorbidities, and mobility level of the assisted person. Post hoc pairwise comparisons were conducted using the Bonferroni correction to adjust significance levels and control for type I error when significant differences were identified. In addition, partial nonparametric correlations were computed to examine the associations between caregiver burden, HRQoL, and caregiver engagement while adjusting for potential confounders (age, sex, country, cohabitation status, daily caregiving time, number of comorbidities, and the mobility level of the assisted person). Since SPSS does not provide a direct procedure for partial Spearman correlations, all variables of interest and covariates were first rank-transformed, and partial correlation analyses were subsequently conducted on the ranked data. This procedure corresponds to a partial Spearman correlation, as it preserves the ordinal nature of the data while controlling covariates.^29^ The correlation strength (*r*) was categorized as follows: *r* > 0.7 as strong, 0.3 < *r* < 0.7 as moderate, and *r* < 0.3 as weak. Additionally, caregiver engagement scores were dichotomized into low and high-engagement levels (cutoff scores <3 and ≥3, respectively), and group differences were analyzed using the Mann–Whitney *U* test, with EQ-5D-3L and caregiver burden scores serving as comparative measures. This categorization enabled the exploration of engagement as a psychosocial differentiator of caregiving outcomes, consistent with the theoretical premise that engagement moderates the impact of caregiving stressors on well-being. All tests were 2-tailed, with statistical significance defined as *P* <.05.

### Ethics Approval and Consent to Participate

Ethical approval from local and national authorities was sought and granted in Italy (Regione Del Veneto, Azienda Ospedale—Università Padova, Comitato Etico per la Sperimentazione Clinica della Provincia di Padova, ref. 5308/AO/22), Spain (Ethics Committee of Virgen de la Arrixaca University Hospital, ref. 3801/2022), and Romania (Comisia Naţională de Bioetică a Medicamentului şi a Dispozitivelor Medicale, ref. 2SNI/13.01.2022). All participants provided informed consent. On the initial page of the online survey, participants were required to provide informed consent, which was necessary to access the primary survey. All data were anonymous, ensuring participant confidentiality and adherence to ethical and legal data protection standards.

## Results

### Characteristics of Informal Caregivers

Table 1 outlines the demographic and caregiving profiles of 179 informal caregivers. The median (IQR) age of caregivers was 56.0 (48.0–71.0) years, with almost three-quarters being women (72.6%) and predominantly White (99.4%). Caregivers were recruited from Romania (39.7%), Spain (35.2%), and Italy (25.1%). Regarding the relationship, the assisted people were most often the caregiver’s parent (40.2%) or spouse/partner (29.6%), and nearly half of the informal caregivers (49.2%) lived in the same household as the assisted person. The time commitment for caregiving revealed that 50.3% provided care on a nondaily basis, spending less than 6 hours each time, and the duration of caregiving was predominantly long-term, with 41.1% providing care for 5 or more years.

**TABLE 1:** Characteristics of Informal Caregivers and Assisted People (N = 179)

Characteristics	Median (IQR), n (%)
Age (years)	56.0 (48.0–71.0)
18–44	35 (19.6)
45–64	86 (48.0)
65–79	35 (19.6)
≥80	23 (12.8)
Female	130 (72.6)
Ethnicity	
White	178 (99.4)
Other	2 (0.6)
Country	
Romania	71 (39.7)
Spain	63 (35.2)
Italy	45 (25.1)
Educational attainment	
Primary school	24 (13.4)
Secondary school	72 (40.2)
Degree level or above	71 (39.7)
Other/prefer not to say	12 (6.7)
Relationship with the assisted person	
Parent	72 (40.2)
Spouse/partner	53 (29.6)
Any other relative	51 (28.5)
Friend	3 (1.7)
Cohabitant with the assisted person	88 (49.2)
Daily caregiving time commitment	
Full-time	37 (20.7)
Less than 6 hour/day, Daily	52 (29.1)
Less than 6 hour/day, Nondaily	90 (50.3)
Duration of caregiving (years)	
≤1	49 (27.4)
2–4	51 (28.5)
≥5	79 (44.1)
Number of comorbidities of the assisted person	
1–2	78 (43.6)
3–5	75 (41.9)
>5	26 (14.5)
Existing comorbidities of the assisted person^a^	
Heart disease	154 (86.0)
High blood pressure	115 (64.2)
Diabetes	63 (35.2)
Vision problems	39 (21.8)
Hearing problems	39 (21.8)
Osteoarthritis	38 (21.2)
Thyroid problems	37 (20.7)
Kidney disease	36 (20.1)
Gastrointestinal diseases	31 (17.3)
Previous stroke	26 (14.5)
Cognitive decline	25 (14.0)
Chronic obstructive pulmonary disease	21 (11.7)
Cancer	21 (11.7)
Osteoporosis/previous hip fracture	20 (11.2)
Chronic pain	17 (9.5)
Rheumatoid arthritis	12 (6.7)
Dementia	7 (3.9)
Chronic liver disease	6 (3.4)
Parkinson’s disease	5 (2.8)
Multiple sclerosis	1 (0.6)
Number of medications taken by the assisted person	
None	5 (2.8)
1–2	7 (3.9)
3–4	40 (22.3)
≥5	127 (70.9)
Mobility level of the assisted person	
Can walk independently	114 (63.7)
Walks with a cane/walking stick	31 (17.3)
Walks with a walker/Zimmer frame	24 (13.4)
Moves around with a wheelchair	4 (2.2)
Confined at home, mostly lying on the bed	6 (3.4)
Daily activities that require assistance^a^	
Transferring	131 (73.2)
Bathing	66 (36.9)
Dressing	35 (19.6)
Toileting	23 (12.8)
Eating	27 (15.1)

Abbreviation: IQR, interquartile range.

a Participants reported more than one response.

The assisted individuals frequently had multiple comorbidities and polypharmacy: 43.6% had 1 or 2 comorbidities, with heart diseases (86.0%) and hypertension (64.2%), and most (70.9%) were on 5 or more medications. Regarding the mobility levels of the assisted persons, 63.7% could walk independently, and the daily activities requiring assistance mainly included transferring (73.2%) and bathing (36.9%).

### Levels of Caregiver Burden, HRQoL, and Caregiver Engagement

The perceived burden was moderate for the overall sample (median: 62.0, IQR: 52.0–73.0). Regarding the EQ-5D-3L scores, informal caregivers reported minimal impairment in mobility (median: 1.0, IQR: 1.0–1.0), self-care (median: 1.0, IQR: 1.0–1.0), and usual activities (median: 1.0, IQR: 1.0–1.0), though they experienced mild to moderate pain/discomfort (median: 1.0, IQR: 1.0–2.0) and anxiety/depression (median: 1.0, IQR: 1.0–2.0). Participants also reported a relatively high level of perceived overall health (median: 80.0, IQR: 55.0–90.0) on the VAS. The median score (IQR) of caregiving engagement was 3.0 (2.0–3.0), and most participants fell into the hyperactivation (n = 53, 29.6%) or drowning (n = 93, 52.0%) phases. However, only 17.3% (n = 31) achieved the highest phase of caregiver engagement (balance).

### Differences in Caregiver Burden, HRQoL, and Caregiver Engagement Compared by Caregiving Characteristics

Table 2 reports differences in caregiver burden, EQ-5D-3L, and caregiver engagement scores compared by age groups, sex, country, cohabitation with the assisted person, daily caregiving time, duration of caregiving, number of comorbidities, and mobility level of the assisted person. Caregiver burden varied significantly across age groups, with caregivers aged 80 years or older reporting a significantly lower burden than those aged 18 to 44 and 45 to 64 years (*P* < .001). Age-related differences were also observed across several domains of HRQoL. Informal caregivers aged 65 to 79 and those aged 80 and above experienced significantly more mobility limitations than those aged 18 to 44 and 45 to 64 (*P* < .001), with no significant difference between the 2 older groups. Self-care functioning also differed significantly: caregivers aged 80 and above reported greater self-care difficulties than all younger groups, and those aged 65 to 79 had worse scores than those aged 18 to 44 (*P* < .001). In the usual activities domain, caregivers aged 65 to 79 and those aged 80 and above reported more limitations compared with the 18 to 44 group, with the ≥80 group also scoring worse than the 45 to 64 group (*P* < .001). Regarding psychological well-being, caregivers aged older than 80 reported higher levels of anxiety/depression than those aged 18 to 44 (*P* < .05). Moreover, informal caregivers aged 80 and above reported significantly worse overall health compared with those aged 18 to 44 and 45 to 64 (*P* < .05). No significant age-related differences were found in the pain/discomfort domain or caregiver engagement scores.

**TABLE 2:** Differences in Caregiver Burden, Health-Related Quality of Life, and Caregiver Engagement: Comparison by Age Groups, Sex, Country, Cohabitation, Daily Caregiving Time, Duration of Caregiving, Number of Comorbidities, and Mobility Level of the Assisted Person (N = 179)

Variables/Questionnaires Median (IQR)	Caregiver Burden	Health-Related Quality of Life						Caregiver Engagement
		Mobility	Self-Care	Usual Activities	Pain/Discomfort	Anxiety/Depression	Overall Health	
Age groups								
18–44	60.0 (50.0–82.0)	1.0 (1.0–1.0)	1.0 (1.0–1.0)	1.0 (1.0–1.0)	1.0 (1.0–2.0)	1.0 (1.0–2.0)	80.0 (70.0–90.0)	3.0 (2.0–3.0)
45–64	58.5 (50.0–65.0)	1.0 (1.0–1.0)	1.0 (1.0–1.0)	1.0 (1.0–1.0)	1.0 (1.0–2.0)	1.0 (1.0–2.0)	80.0 (50.0–90.0)	3.0 (2.0–3.0)
65–79	64.0 (58.0–73.0)	1.0 (1.0–2.0)^c^	1.0 (1.0–1.0)^e^	1.0 (1.0–2.0)^e^	2.0 (1.0–2.0)	2.0 (1.0–2.0)	70.0 (50.0–80.0)	3.0 (2.0–4.0)
≥80	85.0 (63.0–92.0)^c^	2.0 (1.0–2.0)^c^	1.0 (1.0–2.0)^d^	2.0 (1.0–2.0)^c^	1.0 (1.0–2.0)	2.0 (1.0–2.0)^e^	68.0 (45.0–75.0)^c^	3.0 (2.0–3.0)
*P* value^a^	**<.001**	**<.001**	**<.001**	**<.001**	.245	**.016**	**.003**	.299
Sex								
Female	60.0 (50.0–70.0)	1.0 (1.0–1.0)	1.0 (1.0–1.0)	1.0 (1.0–1.0)	1.0 (1.0–2.0)	2.0 (1.0–2.0)	80.0 (50.0–90.0)	3.0 (2.0–3.0)
Male	64.0 (58.0–86.5)	1.0 (1.0–2.0)	1.0 (1.0–1.0)	1.0 (1.0–1.0)	1.0 (1.0–2.0)	1.0 (1.0–2.0)	75.0 (58.5–90.0)	3.0 (2.0–3.0)
*P* value^b^	**.009**	.159	.220	.905	.069	.065	.948	.612
Country								
Italy	60.0 (52.5–64.0)	1.0 (1.0–2.0)	1.0 (1.0–1.0)	1.0 (1.0–2.0)	2.0 (1.0–2.0)	2.0 (1.0–2.0)	75.0 (50.0–87.5)	3.0 (2.0–3.0)
Romania	58.0 (49.0–70.0)	1.0 (1.0–1.0)	1.0 (1.0–1.0)^g^	1.0 (1.0–1.0)	1.0 (1.0–2.0)	1.0 (1.0–2.0)^h^	80.0 (70.0–90.0)^g^	3.0 (2.0–3.0)
Spain	66.0 (62.0–88.0)^f^	1.0 (1.0–2.0)	1.0 (1.0–1.0)	1.0 (1.0–2.0)	1.0 (1.0–2.0)	1.0 (1.0–2.0)	70.0 (50.0–87.0)	3.0 (2.0–3.2)^h^
*P* value^a^	**<.001**	.070	**.007**	.272	.244	**.040**	**.007**	**.018**
Cohabitation with the assisted person								
No	60.5 (52.0–66.0)	1.0 (1.0–1.0)	1.0 (1.0–1.0)	1.0 (1.0–1.0)	1.0 (1.0–2.0)	1.0 (1.0–2.0)	80.0 (70.0–90.0)	3.0 (2.0–3.0)
Yes	63.5 (52.3–79.8)	1.0 (1.0–2.0)	1.0 (1.0–1.0)	1.0 (1.0–2.0)	1.0 (1.0–2.0)	2.0 (1.0–2.0)	70.0 (50.0–85.0)	3.0 (2.0–3.0)
*P* value^b^	.132	.076	.143	.069	.203	**.034**	**.013**	762
Daily caregiving time								
Full-time	71.0 (56.5–88.0)^i^	1.0 (1.0–2.0)	1.0 (1.0–2.0)	1.0 (1.0–2.0)	1.0 (1.0–2.0)	2.0 (1.0–2.0)	65.0 (47.5–80.0)	3.0 (2.0–3.5)
Less than 6 hour/day, Daily	60.0 (44.8–77.3)	1.0 (1.0–2.0)	1.0 (1.0–1.0)	1.0 (1.0–2.0)	1.0 (1.0–2.0)	2.0 (1.0–2.0)	70.0 (50.0–86.8)	3.0 (2.0–3.0)
Less than 6 hour/day, Nondaily	61.0 (54.8–65.0)	1.0 (1.0–1.0)^j^	1.0 (1.0–1.0)^j^	1.0 (1.0–1.0)^j^	1.0 (1.0–2.0)	1.0 (1.0–2.0)^j^	80.0 (70.0–90.0)^k^	3.0 (2.0–3.0)
*P* value^a^	**.025**	**.002**	**.011**	**.008**	.728	**.002**	**<.001**	.490
Duration of caregiving (years)								
≤1	62.0 (51.0–73.0)	1.0 (1.0–1.0)	1.0 (1.0–1.0)	1.0 (1.0–1.0)	1.0 (1.0–2.0)	1.0 (1.0–2.0)	80.0 (50.0–90.0)	3.0 (2.0–3.0)
2–4	62.0 (55.0–85.0)	1.0 (1.0–2.0)	1.0 (1.0–1.0)	1.0 (1.0–2.0)	1.0 (1.0–2.0)	1.0 (1.0–2.0)	70.0 (55.0–86.0)	3.0 (2.0–3.0)
≥5	59.0 (50.0–66.0)	1.0 (1.0–1.0)	1.0 (1.0–1.0)	1.0 (1.0–1.0)	1.0 (1.0–2.0)	2.0 (1.0–2.0)	80.0 (50.0–90.0)	3.0 (2.0–3.0)
*P* value^a^	.206	.084	.257	.725	.221	.250	.767	.787
Number of comorbidities								
1–2	62.0 (50.0–67.0)	1.0 (1.0–1.0)	1.0 (1.0–1.0)	1.0 (1.0–1.0)	1.0 (1.0–2.0)	1.0 (1.0–2.0)	80.0 (55.3–90.0)	3.0 (2.0–3.0)
3–5	61.0 (53.0–79.0)	1.0 (1.0–1.0)	1.0 (1.0–1.0)	1.0 (1.0–1.0)	1.0 (1.0–2.0)	1.0 (1.0–2.0)	80.0 (60.0–90.0)	3.0 (2.0–3.0)
>5	62.0 (52.0–90.5)	2.0 (1.0–2.0)^l^	1.0 (1.0–2.0)^m^	1.5 (1.0–2.0)^l^	1.0 (1.0–2.0)	2.0 (1.0–2.0)	70.0 (40.0–90.0)	3.0 (2.0–3.0)
*P* value^a^	.635	**.002**	**.014**	**.002**	.297	.079	.252	.732
Mobility level of the assisted person								
Can walk independently	62.0 (53.8–72.0)	1.0 (1.0–1.0)^n^	1.0 (1.0–1.0)	1.0 (1.0–1.0)^n^	1.0 (1.0–2.0)	1.0 (1.0–2.0)	80.0 (60.0–90.0)	3.0 (2.0–3.0)
Walks with a cane/walking stick	65.0 (51.0–85.0)	1.0 (1.0–2.0)	1.0 (1.0–1.0)	1.0 (1.0–2.0)	1.0 (1.0–2.0)	1.0 (1.0–2.0)	70.0 (50.0–85.0)	3.0 (2.0–3.0)
Walks with a walker/Zimmer frame	60.5 (53.3–85.0)	1.0 (1.0–2.0)	1.0 (1.0–2.0)	1.0 (1.0–2.0)	1.0 (1.0–2.0)	2.0 (1.0–2.0)	69.0 (40.0–93.8)	3.0 (2.0–3.0)
Moves around with a wheelchair	77.5 (55.3–106.5)	1.5 (1.0–2.0)	1.0 (1.0–1.8)	1.5 (1.0–2.0)	1.0 (1.0–1.8)	1.5 (1.0–2.0)	89.0 (57.5–99.5)	3.5 (2.3–4.0)
Confined at home, mostly lying on the bed	49.0 (38.8–55.8)	2.0 (1.0–2.0)	1.0 (1.0–1.3)	1.5 (1.0–2.0)	2.0 (1.8–2.0)	2.0 (1.8–2.0)	80.0 (50.0–91.3)	2.0 (2.0–2.3)
*P* value^a^	.061	**<.001**	.116	**.020**	.292	.441	.101	.257

Bold-faced values indicate *P* <.05 was statistically significant.

Abbreviation: IQR; interquartile range.

a Kruskal–Wallis test was performed.

b Mann–Whitney *U* test was performed.

c Significantly different compared with “18–44” and “45–64” groups.

d Significantly different compared with “18–44,” “45–64,” and “65–79” groups.

e Significantly different compared with “18–44” group.

f Significantly different compared with Italy and Romania.

g Significantly different compared with Spain.

h Significantly different compared with Italy.

i Significantly different compared with “Less than 6 hour/day, daily” and “Less than 6 hour/day, non daily” groups.

j Significantly different compared with “Full-time” group.

k Significantly different compared with “Full-time” and “Less than 6 hour/day, daily” groups.

l Significantly different compared with “1–2” and “3–5” groups.

m Significantly different compared with “1–2” group.

n Significantly different compared with “Walks with a cane/walking stick,” “Walks with a walker/Zimmer frame,” and “Confined at home, mostly lying on the bed” groups.

Sex-based differences were observed in caregiver burden, with female caregivers reporting significantly greater burden than male caregivers (*P* < .05). However, no significant differences emerged between males and females across the HRQoL domains—including mobility, self-care, usual activities, pain/discomfort, anxiety/depression, and perceived overall health—or in caregiver engagement scores (all *P* > .05).

Informal caregivers of patients with AF from Spain reported a lower caregiver burden than caregivers from Italy and Romania (*P* < .001). Regarding HRQoL, caregivers from Romania reported significantly better self-care compared with caregivers from Spain and lower anxiety/depression compared with caregivers from Italy (*P* < .001). The overall health status was significantly higher among Romanian caregivers than among those from Spain and Italy (*P* < .001). However, there were no significant differences in mobility, usual activities, and pain/discomfort domains of HRQoL. Preliminary analyses indicated a significant overall difference in caregivers’ engagement between countries. The caregiver engagement was significantly higher among Spanish caregivers compared with the participants from Italy (*P* < .001).

Caregiver burden and engagement did not significantly differ based on whether the caregiver cohabited with the assisted person (*P* > .05). However, significant differences emerged in specific HRQoL domains: caregivers living with the assisted person reported significantly more pain and discomfort, as well as lower perceived overall health, compared with noncohabiting caregivers (*P* < .05). No significant differences were found in mobility, self-care, usual activities, or anxiety/depression domains.

Regarding daily caregiving time, full-time caregivers reported a lower burden compared with the daily and nondaily caregiver groups (*P* < .001). Based on the HRQoL levels, nondaily caregivers reported significantly better scores in mobility, self-care, and usual activities than full-time caregivers. Nondaily caregivers also experienced less anxiety/depression compared with full-time caregivers (*P* < .001). Notably, overall health status scores were significantly higher in the nondaily caregiving group compared with the daily and full-time caregiver groups. However, caregiver engagement scores did not differ significantly in terms of daily caregiving time.

Across the caregiving duration categories, findings showed no statistically significant differences in caregiver burden, HRQoL domains, and caregiver engagement scores (*P* > .05). Additionally, significant differences were observed in caregivers’ HRQoL scores based on the number of comorbidities of the person being assisted. Caregivers assisting individuals with more than 5 comorbidities reported significantly worse HRQoL in the mobility and usual activities domains than the other 2 groups, as well as worse self-care compared with the “2 or fewer comorbidities” group. There were no significant differences in caregiver burden, pain/discomfort, anxiety/depression, and perceived overall health domains of HRQoL, as well as caregiver engagement.

The mobility/physical independence level of the assisted person significantly impacted the HRQoL of caregivers. Caregivers of individuals who could walk independently reported significantly greater HRQoL regarding the mobility domain and significantly better usual activities compared with other groups with more limited movement capacity, such as those who require mobility aids or are confined to bed. In contrast, there were no significant differences in caregiver burden, self-care, pain/discomfort, anxiety/depression, perceived overall health domains of HRQoL, and caregiver engagement.

### Partial Correlations Between Caregiver Burden, HRQoL, and Caregiving Engagement

Table 3 demonstrated the partial correlation analysis regarding associations between caregiver burden, EQ-5D-3L dimensions, and caregiver engagement scores. The caregiver burden scores showed a moderate positive correlation with caregiver engagement (*r* = 0.504, *P* < .001) and a moderate negative correlation with the EQ-5D-3L anxiety/depression domain (*r* = −0.436, *P* < .001). Within the EQ-5D-3L domains, mobility correlated moderately with self-care (*r* = 0.511, *P* < .001) and usual activities (*r* = 0.492, *P* < .001), while self-care and usual activities were also moderately interrelated (*r* = 0.648, *P* < .001). Overall health perception showed moderate negative correlations with mobility (*r* = −0.391, *P* < .001), self-care (*r* = −0.334, *P* < .001), usual activities (*r* = −0.436, *P* < .001), pain/discomfort (*r* = −0.438, *P* < .001), and anxiety/depression (*r* = −0.333, *P* < .001). Finally, caregiver engagement exhibited a moderate negative correlation with the anxiety/depression domain of the EQ-5D-3L (*r* = −0.339, *P* < .001).

**TABLE 3:** Partial Correlations Between Caregiver Burden, Health-Related Quality of Life Dimensions, and Caregiver Engagement Scores (N = 179)

	Caregiver Burden	Mobility	Self-Care	Usual Activities	Pain/Discomfort	Anxiety/Depression	Overall Health	Caregiver Engagement
Caregiver burden	-							
Mobility	−0.031	-						
Self-care	0.009	0.511^**^	-					
Usual activities	−0.047	0.492^**^	0.648^**^	-				
Pain/discomfort	−0.241^*^	0.236^*^	0.138	0.202^*^	-			
Anxiety/depression	−0.436^**^	0.138	0.087	0.227^*^	0.307^**^	-		
Overall health	0.201^*^	−0.391^**^	−0.334^**^	−0.436^**^	−0.438^**^	−0.333^**^	-	
Caregiver engagement	0.504^**^	−0.073	−0.016	−0.096	−0.058	−0.329^**^	0.187^*^	-

* *P* <.05 was considered statistically significant.

** *P* <.001 was considered statistically significant.

### Comparison of Low and High Caregiver Engagement Groups by Caregiver Burden and HRQoL Scores

Table 4 compares caregiver burden and HRQoL scores according to caregiver engagement levels. Based on caregiver engagement scores, 124 participants (69.3%) were classified as highly engaged and 55 (31.7%) as low-engaged. Caregivers in the high-engagement group reported significantly lower burden compared with those in the low-engagement group. No significant differences were observed in the HRQoL domains of mobility, self-care, usual activities, or pain/discomfort. In contrast, caregivers in the low-engagement group reported significantly higher anxiety/depression, while those in the high-engagement group reported significantly better perceived overall health.

**TABLE 4.** Informal Caregivers Grouped by High and Low Levels of Engagement According to Their Caregiver Engagement Scores: Comparison by Caregiver Burden and Health-Related Quality of Life (N = 179)

Variables	Caregiver Engagement		*P*-Value
	High (n = 124) (69.3%^a^)	Low (n = 55) (30.7%^a^)	
Caregiver burden^b^, median (IQR)	64.0 (58.0–83.5)	53.0 (44.0–60.0)	**<.001**
Health-related quality of life^b^, median (IQR)			
Mobility	1.0 (1.0–1.8)	1.0 (1.0–2.0)	.982
Self-care	1.0 (1.0–1.0)	1.0 (1.0–1.0)	.816
Usual activities	1.0 (1.0–1.0)	1.0 (1.0–2.0)	.397
Pain/discomfort	1.0 (1.0–2.0)	1.0 (1.0–2.0)	.259
Anxiety/depression	1.0 (1.0–2.0)	2.0 (1.0–2.0)	**<.001**
Overall health	80.0 (60.0–90.0)	70.0 (50.0–85.0)	**.025**

Bold-faced values indicate *P* <.05 was statistically significant.

Abbreviation: IQR, interquartile range.

a Percentage of the total sample.

b Mann–Whitney *U* test was performed.

## Discussion

In the present study, we examined the caregiving experiences of informal caregivers of patients with AF and multimorbidity across 3 European countries. Our findings highlight substantial variability in caregiving outcomes shaped by demographic, contextual, and caregiving-related characteristics. The analyses indicate that informal caregivers reported a moderate level of burden overall and were generally able to maintain functioning in key domains of HRQoL, including mobility, self-care, and usual activities. Nonetheless, emotional and psychological dimensions were more adversely affected, with caregivers frequently reporting pain/discomfort and heightened levels of anxiety and depression. Although some physical disturbances may be age-related, they more likely reflect the physical and psychosomatic strain associated with caregiving duties, such as assisting with mobility or prolonged daily tasks. Caregiver engagement, assessed through the Caregiver Health Engagement Model, revealed that most informal caregivers occupied the hyperactivation or drowning phases, while only a small proportion reached the balance phase. Together, these findings highlight the coexistence of functional resilience and psychological vulnerability, as well as substantial variability in the degree to which informal caregivers adapt to and integrate their role.^6,7,13^

Regarding the age-related patterns, younger and middle-aged informal caregivers reported a higher burden, whereas older caregivers—particularly those aged 80 and above—had lower burden scores, but markedly poorer HRQoL across the domains of mobility, self-care, usual activities, and psychological well-being. This apparent paradox aligns with previous evidence suggesting that older caregivers may appraise caregiving differently, viewing it as a normative life stage responsibility or as less disruptive to career and social activities.^30^ Moreover, the declining physical and mental health of older caregivers likely exacerbates functional impairments, resulting in low HRQoL, suggesting that lower reported burden in this group may underestimate actual strain due to adaptation or resignation processes.^31^ These findings highlight the importance of tailoring support interventions not only to caregiving intensity but also to age-related vulnerabilities and caregiver resources.

Female caregivers reported significantly higher burden than male caregivers, consistent with prior evidence of gendered role strain in caregiving^32^; however, no sex differences were observed in HRQoL or caregiver engagement. These findings align with the broader literature, which shows that women are often more emotionally and practically invested in caregiving tasks, which may amplify their subjective sense of burden.^33^ This suggests that women may perceive caregiving as more emotionally taxing, even when this does not translate into measurable differences in overall health or engagement. Cardiovascular nurses should therefore remain attentive to these gendered patterns, providing psychosocial support that acknowledges women’s subjective burden while also mobilizing family and community resources to reduce role overload.

Cross-national differences underscore the influence of cultural and systemic contexts on informal caregiving experiences. Spanish caregivers reported lower burden and higher engagement, while Romanian caregivers showed better self-care and lower anxiety/depression, and Italian caregivers reported comparatively poorer psychological outcomes. These variations may reflect differing levels of formal service provision, cultural familism, and expectations of caregiving. Within the AF-specific context, such differences likely stem not only from cultural and community norms but also from variations in healthcare infrastructures.^34^ For example, Spain’s decentralized system, with strong primary care networks and integrated community services such as home and community care, may provide more substantial support for caregivers.^35,36^ In contrast, more centralized or under-resourced systems, as in parts of Romania and Italy, may place greater pressure on family members, simultaneously promoting patient self-management while amplifying caregiver strain.^35^ Nonetheless, Romanian caregivers reported better HRQoL in domains such as self-care and anxiety/depression, possibly reflecting adaptive cultural expectations or unmeasured social support. Acknowledging the substantial burden and life changes associated with informal caregiving across diverse cultural contexts is crucial for developing effective support systems. In some cultures, caregiving is viewed as a moral obligation, with strong family-based norms discouraging external assistance; depending on available resources and social structures, this can either mitigate or exacerbate stress.^36^ For instance, in Mediterranean countries, strong familial bonds and cultural expectations emphasize family responsibility in caregiving, often intensifying both emotional and physical demands.^34^ Such diversities resonate with prior evidence that comprehensive social and institutional support, or conversely, reliance on strong family-based traditions, can either buffer or heighten caregiver experiences.^37,38^ While earlier studies have predominantly focused on caregiver burden and psychological distress,^6,12^ our findings extend this evidence by showing that the highest levels of caregiver engagement are not necessarily bound to national contexts or cultural practices but may instead reflect more universal psychosocial factors involved in adapting to the complexities of AF management.^16^

Cohabitation was associated with greater pain/discomfort and lower self-rated health among informal caregivers. Living in the same household often transforms caregiving into a continuous, boundary-less role marked by nighttime vigilance, curtailed recovery, and fewer opportunities for physical activity or respite, consistent with previous evidence linking cohabitation to higher caregiver strain and health decline.^39^ Moreover, cohabiting informal caregivers frequently assume physically demanding activities of daily living such as transfers and bathing, tasks strongly associated with musculoskeletal symptoms and injury risk.^40^ From a nursing standpoint, cohabiting caregivers should be regarded as a priority risk group: proactive screening for musculoskeletal strain, sleep disturbance, and mood symptoms is essential, alongside structured ergonomics and safe-mobility training. Cardiovascular nurses can also play a crucial role in facilitating access to respite services and community supports through integrated primary and home care linkages, which have been shown to mitigate caregiver burden and improve both caregiver and patient outcomes.^41^

An additional insight derived from our findings is that increased caregiving time does not universally equate to a heightened burden in the context of AF and multimorbidity. Full-time informal caregivers who have reorganized their entire schedule around patient care sometimes reported lower perceived burdens than those providing fewer hours of care each day, potentially because this concentrated approach may streamline access to resources, schedules, and clinical routines.^18,42^ This pattern suggests that psychological adaptation (lower burden appraisals) can coexist with cumulative functional costs when self-care time is curtailed and social participation is limited.^18,42–44^ Nevertheless, longer and more intensive caregiving remains associated with reduced HRQoL, particularly in mobility and anxiety/depression domains—reiterating that while some caregivers may adapt to comprehensive care routines, the prolonged stress of managing multiple chronic conditions can eventually exacerbate strain.^45^

Interestingly, caregiving duration was not associated with significant differences in burden, HRQoL, or engagement. This contrasts with prior literature suggesting that longer caregiving durations may exacerbate strain or, alternatively, promote adaptation. Our results may reflect heterogeneity in disease progression, caregiver resources, or personal coping strategies. Similarly, higher levels of patient comorbidity and limited mobility were associated with lower caregiver HRQoL in physical domains but not with burden or engagement. These findings suggest that while objective care demands impact functional health, they may not always translate into subjective distress if caregivers develop effective coping strategies or perceive their roles as meaningful.^16,43,46^

Interestingly, our results showed a moderate negative correlation between caregiver burden and engagement, indicating that highly engaged caregivers tend to feel less burdened and thus better able to preserve their HRQoL.^45^ However, other evidence suggests that burden itself may sometimes act as a catalyst, motivating informal caregivers to seek support and become more engaged.^47^ This dual pathway implies that some caregivers adopt a proactive engagement style to prevent burden escalation, whereas others may engage reactively after reaching a threshold of strain, depending on individual resources, mindset, and disease complexity.^12^ Overall, engaged caregivers are more likely to employ adaptive coping strategies that reduce psychological distress and physical strain, enabling them to sustain their caregiving role without compromising quality of life.^43,45^ Literature further emphasizes the importance of aligning caregiving responsibilities with informal caregivers’ own life priorities to prevent exhaustion and promote sustainability.^6^ In addition, active engagement has been shown to foster emotional well-being, social functioning, and resilience, ultimately enhancing both caregiving effectiveness and personal health outcomes.^44,46^

Finally, the differences between highly engaged caregivers and those with low engagement were pronounced, reinforcing the role of engagement as a potential “buffer” in caregiving for patients with AF who often require multidimensional care. Highly engaged caregivers reported notably lower burden, reduced anxiety/depression, and better perceived overall health, reflecting the protective nature of engagement on coping efficacy and emotional stability.^16,44,46^ This resonates with earlier evidence that robust engagement correlates with higher self-esteem, improved management of caregiving responsibilities, and healthier interpersonal relationships, collectively contributing to a better quality of life.^48,49^ In contrast, less engaged caregivers often experience higher levels of burden, heightened emotional distress, and fragmented coping strategies—exacerbating the already formidable challenge of managing AF with multimorbidity.^6,50^ Consequently, there is a pronounced need for psychosocial interventions, training programs, and policy initiatives that systematically foster engagement by enhancing caregivers’ communication skills, strengthening problem-solving abilities, and establishing supportive clinical community networks.^6^ Through such comprehensive strategies, the additional pressures from AF and coexisting chronic conditions can be more effectively mitigated, leading to better outcomes not only for patients but also for the vital family members entrusted with their care.

Despite the valuable contributions of the current study, several limitations warrant consideration. First, the cross-sectional design restricts causal inferences on how caregiver burden, HRQoL, and engagement evolve or influence one another over time, highlighting the need for longitudinal approaches to capture dynamic shifts in caregiving demands and psychosocial adaptation. Second, reliance on self-reported data may introduce response biases; although the instruments used exhibit strong psychometric properties, subjective measures can be influenced by individual perceptions and situational factors. Third, although the sample encompassed diverse recruitment channels and multiple European countries, the overall size may limit the generalizability of findings, especially within subgroups (eg, culturally distinct populations or caregivers with highly specialized needs). Finally, potential confounders such as variations in healthcare access, socioeconomic status, specific disease severity in multimorbidity, or differing social and family support systems across participating countries were not exhaustively controlled, meriting further research to disentangle how these contextual elements shape the caregiving experience. Future investigations incorporating qualitative and longitudinal methods could deepen insights into the temporal unfolding of caregiver engagement and elucidate culturally tailored strategies to support caregivers in these high-demand settings.

## Conclusions

This study provides a nuanced understanding of the caregiving experience for patients with AF and multimorbidity across 3 European countries. Although informal caregivers commonly reported moderate burden and generally maintained core HRQoL dimensions, they still faced considerable anxiety/depression and risk of burnout, especially when managing multiple chronic conditions. Our findings demonstrate that AF with multimorbidity does not invariably lead to uniform increases in burden or deteriorated quality of life. Higher levels of caregiver engagement were associated with reduced caregiver burden, improved perceived overall health, and fewer symptoms of anxiety and depression. Tailored interventions that bolster caregiver engagement—through role-specific education, robust problem-solving strategies, and consistent healthcare collaboration—may mitigate caregiver strain and promote higher HRQoL in this population.

## Acknowledgments

The authors would like to thank all participants for their contribution and acknowledge the biostatistician’s review during the process.

AFFIRMO Project consortium members are as follows: Søren Påske Johnsen, Aalborg Universitet (AAU), Denmark. Gregory Lip, The University of Liverpool (UOL), United Kingdom. Mirko Petrovic, Universiteit Gent (UGent), Belgium. Davide Liborio Vetrano, Karolinska Institutet (KI), Sweden. Stefania Maggi, Consiglio Nazionale delle Ricerche (CNR), Italy. Gheorghe-Andrei Dan, Universitatea de Medicina si Farmacie “Carol Davila” din Bucuresti (UMFCD), Romania. Nicola Ferri, Università degli Studi di Padova (UNIPD), Italy. Tatjana Potpara, Faculty of Medicine, University of Belgrade (MFUB), Serbia. Laura Vivani, Moverim Consulting sprl (MOVERIM), Belgium. Alessandro Ferri, Advice Pharma Group srl (AdvPha), Italy. Jacek Marczyk, Ontonix, Italy. Trudie Lobban, Arrhythmia Alliance (A-A), United Kingdom. Georg Ruppe, European Union Geriatric Medicine Society aisbl (EuGMS), Belgium. Graziano Onder, Istituto Superiore di Sanità (ISS), Italy. Guendalina Graffigna, Università Cattolica del Sacro Cuore (UCSC), Italy. Aldo Pietro Maggioni, Heart Care Foundation Onlus (HCF), Italy. Dipak Kalra, The European Institute for Innovation through Health Data (I-HD), Belgium. John Ainsworth, The University of Manchester (UNIMAN), United Kingdom. Francisco Marín Ortuño, Universidad De Murcia (UMU), Spain. Mariya Tokmakova, Medical University Plovdiv (MUP), Bulgaria.
